# The use of temporary internal distraction rods for the correction of severe scliosis

**DOI:** 10.1186/1748-7161-10-S1-P25

**Published:** 2015-01-19

**Authors:** Masatoshi Inoue, Hidehisa Torikai, Yoshinori Nakata

**Affiliations:** 1 Chiba Saisei-kai Narashino Hospial, Japan; 2 Nakata Orthopedic Clinic, Japan

## Purpose

Managing patients with scoliosis with curve > 90 degrees remains challenging. Bruchowski et al reported temporary internal distraction rods (TIDRs) can be used instead of halo traction for severe spinal defmormity. We report 5 cases of severe scoliosis managed with a 2-stage operation using a TIDR.

## Patients and methods

Since 2009, 5 patients were treated with spinal instrumentation with TIDRs. These were 4 female and 1 male patients with a mean age of 28 years (13-55 years) at surgery. Mean preoperative Cobb angle was 116 degrees. All patients had restricted pulmonary dysfunction. For all patients, 2-stage operation using temporary internal distraction was performed. In one case, anterior release followed by 2-stage operation with a temporary distraction rod was performed.

## Results

Mean postoperative Cobb angle was 39 degrees (22-67 degrees), corresponding to a mean correction rate of 69% (55-75 %). Mean estimated blood loss was 4024ml, but only one patient required homologous blood transfusion. After first stage operation, CT revealed screw malposition in one patient, but it was removed in 2nd stage operation. We encountered no preoperative complications.

## Discussion

TIDR is an alternative approach in patients undergoing spinal fusion for severe scoliosis. Gradual correction of the spine in 2-stage operation may prevent neurological complications. It decreased the need of homologous blood transfusion.

